# Bone lengthening: bridging joints, soft tissue releases, physiotherapy

**DOI:** 10.1007/s11832-016-0783-z

**Published:** 2016-11-08

**Authors:** Franck Launay, Sébastien Pesenti

**Affiliations:** Service des Urgences Pédiatriques, Hôpital Timone Enfants, 264 Rue St-Pierre, 13005 Marseille, France

**Keywords:** Lengthening, Physiotherapy, Joint, Contracture, Bridging

## Abstract

When we lengthen a bone in a child, the parents and the family circle are often obsessed by the amount a lengthening obtained. However, for the surgeon, lengthen a bone is quite pretty easy, but dealing with the joints above and below the lengthening area can be very challenging. Indeed, during the lengthening process, muscles and tendons will be progressively stretched, leading to potential joint contracture or even dislocation. The objective of the surgeon will be to avoid this situation. The first mean at disposal is the physiotherapy in order to help the joints to be more supple and to maintain their range of motion. The second mean is the soft tissue release before the surgery, during the lengthening process, or after the hardware removal when the capacities of physiotherapy are overdone. As a last resort, it can be helpful to bridge the joint to protect it during the lengthening.

Nowadays, with the advent of modern external fixators such as hexapodal fixators, it is not really a big deal to lengthen a straight bone or to correct a bone deformity. In fact, the most difficult thing is to deal everything around the lengthening such as the growth plate above and below the lengthening zone, the joint above and below the lengthening zone, soft tissues contractures, pin care physiotherapy, family organization, and even school organization.

Concerning the joint above and below the lengthening zone, we have to know that lengthen a bone will lead to joint contracture such as hip flexion contracture, hip adduction contracture, knee flexion contracture, or equinus [1, 2]. In some extreme cases, a dislocation can occur. Thus, we have to think about soft tissues and joints even before applying the hardware on the patient.

In the hip, it is possible to do adductor tenotomies in case of limited hip abduction. In some cases, shelf acetabuloplasty or proximal femur osteotomy can be required. However, by doing this way, we have to do two surgeries: the first one to lengthen the adductor tendons and the second one to lengthen the femur. If we prefer to do a one step surgery, it is crucial to add an intensive physiotherapy programme, because the progressive hip adduction contracture can easily lead to lateral dislocation of the hip. In order to avoid this complication, one can bridge the hip joint with the external fixator, but there is a neat way to avoid this procedure. Indeed, one can use a circular frame to do the femoral lengthening, and the solution is to use a proximal full ring in order to obtain a Milwaukee effect. This effect is based on the principle of spine auto-elongation with the Milwaukee brace in scoliosis treatment. Thus, if a beginning of a hip adduction contracture occurs during the lengthening phase, the patient will have a perineal discomfort, and he will have to put his hip in abduction to avoid any contact between the proximal ring and the perineal zone. So, when the patient complaints about that, one can slow the distraction rate and intensify the physiotherapy programme. This procedure can be enough if it is done early, avoiding the likely tenotomy.

Concerning the knee, it can be helpful to stabilize it if the knee has a multidirectional laxity before doing any femoral or tibial lengthening. But it is also important to check the potential stiffness of the harmstrings and of the rectus femoris with the assessment of the popliteal angle and with the Ely test. In case of stiffness, one have to release the rectus femoris from the anterior inferior iliac spine and/or one have to lengthen the harmstrings distally. Plus, one can recommend to release the fascia lata distally at the level of the proximal pole of the patella if one have to lengthen a congenital short femur in order to avoid any lateral patellar dislocation during the lengthening phase.

Concerning the ankle, it is recommended to lengthen the Achileus tendon to avoid any contracture in equinus during the lengthening phase. But, when this procedure is needed, it is preferable to do it in a separate surgical procedure before applying the hardware.

However, more than soft tissue release during or before the application of the hardware, it can be helpful to bridge the joint. Concerning the hip, it is preferable to let the joint free and to do some soft tissue release, or to apply the principle of the Milwaukee effect. Even if the fact of bridging the hip can be useful in some indications of femoral lengthening, its main indication is for the Legg-Perthes-Calve disease in children with a beginning of a femoral head deformation [3]. The goal of this construction is to distract the hip and to move the femur to abduction, allowing flexion–extension range of motion, in order to bring the femoral head inside the acetabulum and in order to allow a femoral head remodelling (Fig. 1).

**Fig. 1:**
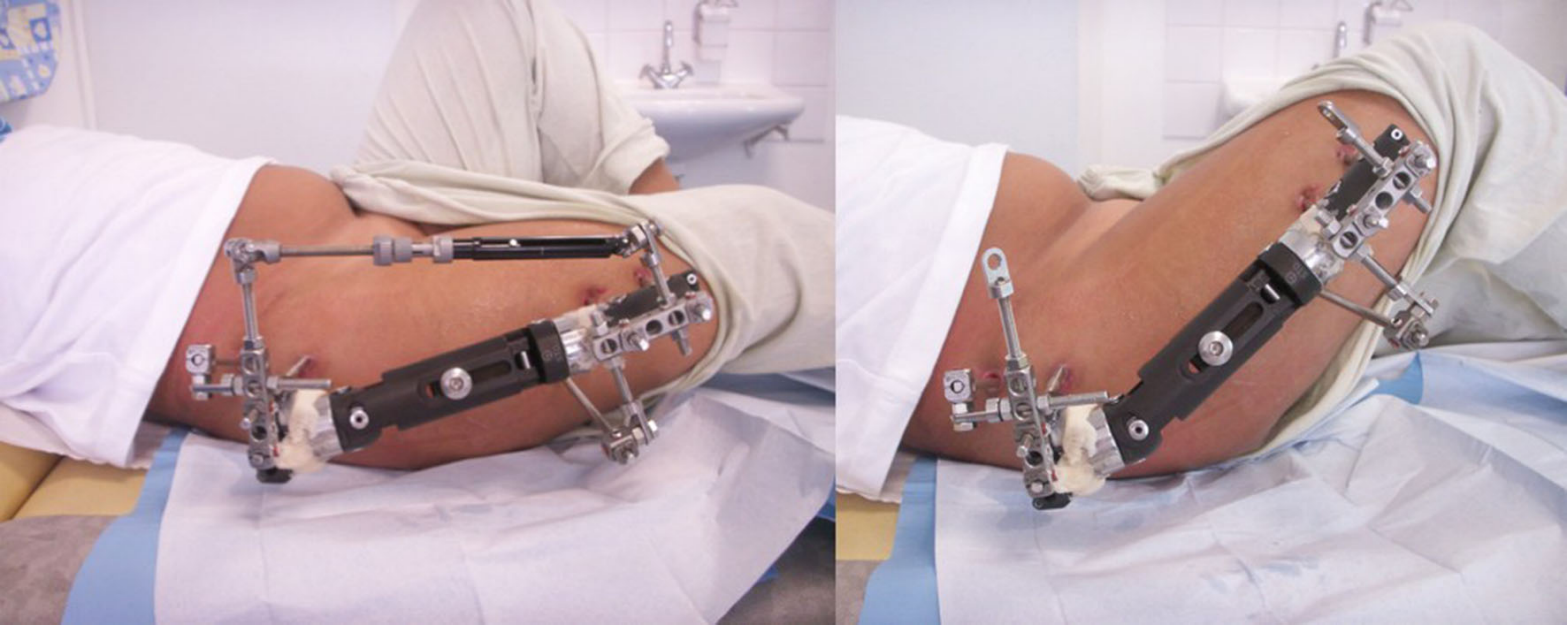
An external fixator can bridge the hip and distract it in case of Legg-Perthes-Calve disease. The construction can allow hip motion in flexion and in extension

Concerning the necessity for bridging the knee, there is more risk of knee contracture when one lengthen a femur in place of the tibia. Thus, it is not necessary to bridge the knee in a tibial lengthening even in case of anterior cruciate ligament deficiency in fibular hemimelia. But it seems to be important to bridge the knee in case of femoral lengthening in a congenital condition. However, bridging the joint is not only lengthening the fixator down to the tibia and blocking the knee. Indeed, if we do that, we won’t allow knee mobilization during several months and it will be very hard to recover all range of motion after that. So, if we want to bridge the knee, we can do it with hinges to allow knee motion. However, this is not the only problem with the fact of bridging the knee. Indeed, we have to realize that when we do that, we bridge not only the knee joint but also two growth plates. Thus, this part of the external fixator will behave as a huge growth modulation staple and will slow the growth down. So, to avoid this kind of problem, we have to distract the knee to allow the natural growth of both physis. An attractive option consists in bridging the knee with a hexapodal fixator and leaving the struts free. Thus, this can allow natural growth of the knee as well as knee motion and active physiotherapy by removing the struts from the rings (Fig. 2). Plus, in case of a beginning of contracture, one can block the system, put a joint distraction, and correct gradually the flexion contracture with the software.

**Fig. 2:**
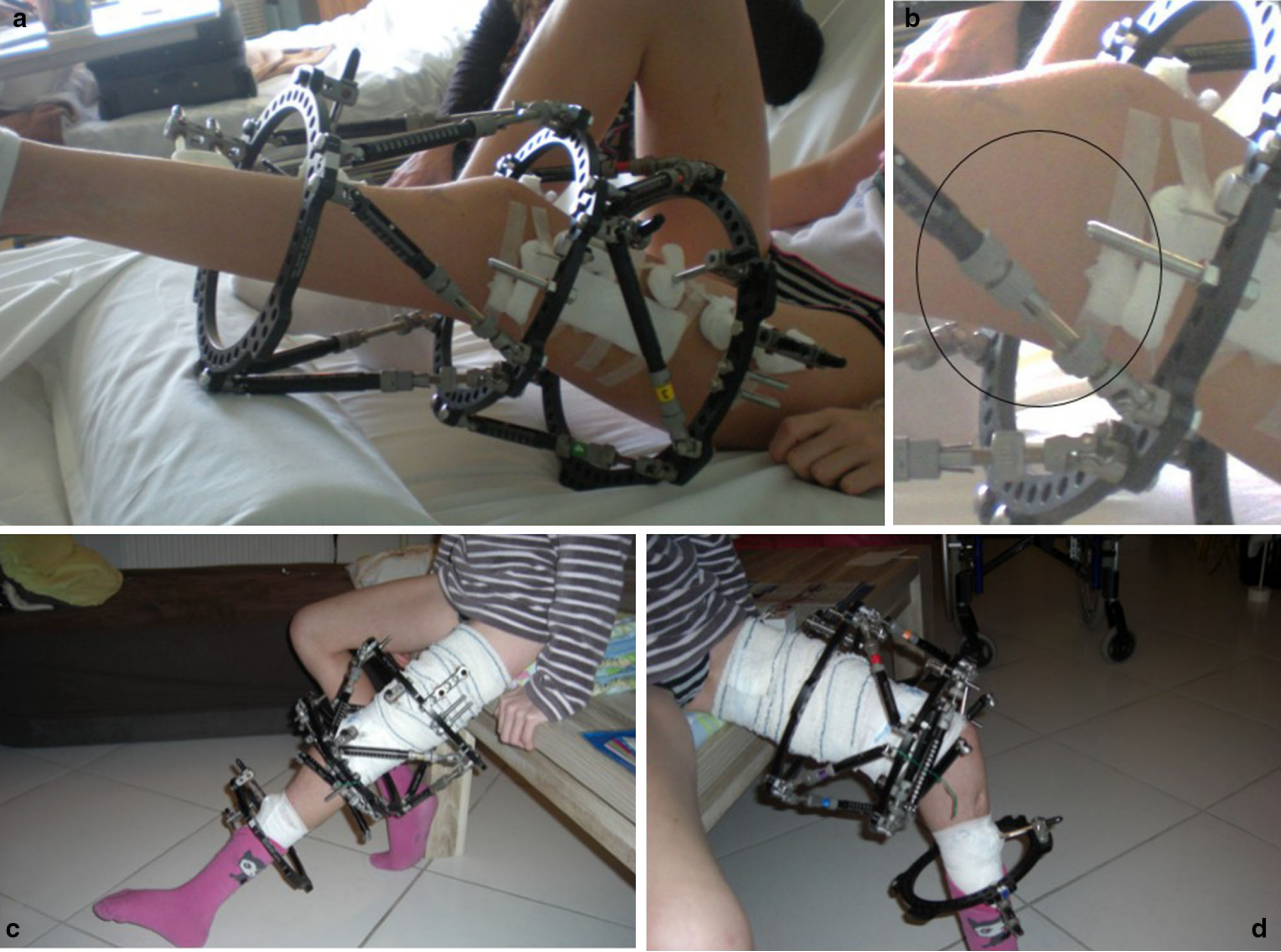
One can bridge the knee with a hexapod fixator **a** but with the struts let free **b** in order to avoid growth modulation. Then, the struts can be removed for physiotherapy allowing knee motion (**c**, **d**)

Finally, concerning the necessity for bridging the ankle, it seems better to avoid it as far as possible, because the patient will walk more easily and better on his own feet instead of on a foot plate, which is better for loads transfer and bone healing. So, in case of a simple decreased dorsiflexion of the ankle, it is better to do a percutaneous tenotomy of the Achileus tendon prior to the surgery associated with brace and active physiotherapy.

Lengthen a bone can be technically very simple but there are so many things to think about around the lengthening, and the most important thing is probably the physiotherapy. The role of the physiotherapist is to stimulate weight bearing and to avoid contracture of the joints during the lengthening process but also to improve the range of motion of joints around the lengthened bone before the surgery. On the physiotherapy depends the outcome of the lengthening in term of range of motion and in term of bone healing. The role of everyone who is involved in the lengthening process is to stimulate daily activities by helping the child and the family to forget the external fixator.
